# Elucidating regulatory processes of intense physical activity by multi-omics analysis

**DOI:** 10.1186/s40779-023-00477-5

**Published:** 2023-10-18

**Authors:** Ernesto S. Nakayasu, Marina A. Gritsenko, Young-Mo Kim, Jennifer E. Kyle, Kelly G. Stratton, Carrie D. Nicora, Nathalie Munoz, Kathleen M. Navarro, Daniel Claborne, Yuqian Gao, Karl K. Weitz, Vanessa L. Paurus, Kent J. Bloodsworth, Kelsey A. Allen, Lisa M. Bramer, Fernando Montes, Kathleen A. Clark, Grant Tietje, Justin Teeguarden, Kristin E. Burnum-Johnson

**Affiliations:** 1 Biological Sciences Division, Pacific Northwest National Laboratory, 902 Battelle Boulevard, Richland, WA 99352 USA; 2 Environmental and Molecular Sciences Division, Pacific Northwest National Laboratory, 902 Battelle Boulevard, Richland, WA 99352 USA; 3 Centers for Disease Control and Prevention, National Institute for Occupational Safety and Health, Western States Division, Denver, CO 80204 USA; 4 Computational Analytics Division, Pacific Northwest National Laboratory, Richland, WA 99352 USA; 5 National Security Directorate, Pacific Northwest National Laboratory, Richland, WA 99352 USA; 6 Los Angeles County Fire Department, Los Angeles, CA 90063 USA; 7 Centers for Disease Control and Prevention, National Institute for Occupational Safety and Health, Respiratory Health Division, Morgantown, WV 26505 USA; 8 Environmental and Molecular Toxicology, Oregon State University, Corvallis, OR 97331 USA

**Keywords:** Multi-omics analysis, Intense exercise, Human performance, Biofluids, Metabolism, Immunity

## Abstract

**Background:**

Physiological and biochemical processes across tissues of the body are regulated in response to the high demands of intense physical activity in several occupations, such as firefighting, law enforcement, military, and sports. A better understanding of such processes can ultimately help improve human performance and prevent illnesses in the work environment.

**Methods:**

To study regulatory processes in intense physical activity simulating real-life conditions, we performed a multi-omics analysis of three biofluids (blood plasma, urine, and saliva) collected from 11 wildland firefighters before and after a 45 min, intense exercise regimen. Omics profiles post- versus pre-exercise were compared by Student’s *t*-test followed by pathway analysis and comparison between the different omics modalities.

**Results:**

Our multi-omics analysis identified and quantified 3835 proteins, 730 lipids and 182 metabolites combining the 3 different types of samples. The blood plasma analysis revealed signatures of tissue damage and acute repair response accompanied by enhanced carbon metabolism to meet energy demands. The urine analysis showed a strong, concomitant regulation of 6 out of 8 identified proteins from the renin-angiotensin system supporting increased excretion of catabolites, reabsorption of nutrients and maintenance of fluid balance. In saliva, we observed a decrease in 3 pro-inflammatory cytokines and an increase in 8 antimicrobial peptides. A systematic literature review identified 6 papers that support an altered susceptibility to respiratory infection.

**Conclusion:**

This study shows simultaneous regulatory signatures in biofluids indicative of homeostatic maintenance during intense physical activity with possible effects on increased infection susceptibility, suggesting that caution against respiratory diseases could benefit workers on highly physical demanding jobs.

**Supplementary Information:**

The online version contains supplementary material available at 10.1186/s40779-023-00477-5.

## Background

Physical activity induces a multi-organ response to increased energy needs, oxygen demands, and the consequences of tissue damage [1]. Demands that exceed adaptive capacity, can overcome these homeostatic mechanisms, leading to exhaustion, decreased human performance, and increased worker susceptibility to injury or disease [2–4]. Improving our understanding of these synchronized regulatory processes would enable us to monitor the balance between adaptive and adverse responses to intense physical exercise to predict the early stages of exhaustion, develop interventions that improve performance and recovery, and mitigate health risks for occupations involving intense physical activity, such as firefighting, law enforcement, military, and sports.

Mass spectrometry-based multi-omics analysis of biological fluids, such as blood plasma, saliva, and urine, is one of the most promising approaches for studying the dynamics of the molecular regulation of normal, adaptive, and disease states in humans. Continual advances in instrumentation have enabled the identification and quantitation of thousands of biomolecules per sample, leading to comprehensive views of the regulated processes [5–10]. A pioneer metabolomics study reported that glycogenolysis, the tricarboxylic acid (TCA) cycle and lipolysis were major activated processes in acute and prolonged aerobic exercises [11]. Contrepois et al. [5] performed a multi-omics analysis of plasma and leukocytes from human volunteers that underwent an acute aerobic exercise session that revealed a highly orchestrated landscape of molecular signals and processes that support human physical activity. Omics analysis has also shown memory improvement due to exercise-mediated anti-inflammatory effects [12]. A meta-omics analysis of elite marathon runners found that the gut bacterium *Veillonella atypica* (*V. atypica*) was associated with increased performance [13]. Additionally, the administration of *V. atypica* to mice improved their performance, which was attributed to the conversion of lactate produced in the muscle into propionate at the intestinal surface [13]. In another example, injection of glycosylphosphatidylinositol-specific phospholipase D1 into sedentary mice was shown to recapitulate the cognitive benefits induced by exercise [14]. These findings represent a proof of concept that physical performance can be improved with exogenous treatments based on an understanding of the underlying physiological processes and their molecular transducers.

A fundamental question in exercise physiology is how different tissues of the body are regulated to meet the physiological and metabolic demands of intense exercise while simultaneously maintaining homeostasis. Here, we investigated the molecular regulatory signatures of strenuous exercise by performing proteomics, lipidomics and metabolomics analyses of wildland firefighters following an intense 45 min exercise session at the aerobic threshold. We chose to study wildland firefighters due to their arduous work under difficult conditions in remote locations for shifts often longer than 24 h, and for up to 14–21 days. During the peak of the 2020 wildfire season (September 2020), over 32,000 wildland fire personnel were deployed across the Western United States to participate in fire suppression [15]. Our data shows strong signatures of molecular regulation in the different biofluids, providing insights into the molecular coordination between tissues during intense physical activity. We also performed a systematic literature review to investigate the potential health consequences of the molecular regulation in response to extreme physical activity and training. This is an important step towards building the capability to monitor the balance between adaptive and adverse responses and to predict the early biochemical and physiological stages of exhaustion, in pursuit of reducing the occupational risk of firefighters, first responders and other high-stress occupations.

## Methods

### Study design, sample cohort and randomization

Sample size calculations were performed to determine the necessary number of subjects assuming an experimental design with data collection before and after exercise. As is standard, a type 1 error rate of 0.05 and a power of 0.80 were assumed, and the median standard deviation, was used to estimate typical variability in the data. Power analyses were conducted based on an ongoing study’s lipidomics dataset, showing that 5 samples would be needed for a 0.8 power to reach statistical significance with ≥ 1.5-fold change in 75% of the measured molecules. A total of 13 male firefighter volunteers with the average age of (25 ± 3) years old and average body mass index of (26.3 ± 3.3) kg/m^2^ before exercise were recruited. All volunteers that agreed to participate were included, without exclusion criteria, to have a better representation of the firefighter population.

The volunteers underwent an exercise session that consisted of hiking outdoors over hilly terrain in Santa Clarita, CA, with full wildland firefighter gear that weighed from 9 to 20 kg. Volunteers’ blood was collected pre- and post-exercise by phlebotomy and immediately placed on ice. The post-sample was collected within 10 min after the exercise. Blood was drawn by professional phlebotomists from different arms for baseline and after exercise sampling to avoid local responses related to the drawing process. The skin surface was wiped with antiseptic tissue before the puncture to minimize contamination. Blood was drawn into one 6 ml ethylenediaminetetraacetic acid (EDTA)-coated vacuum tube (Vacuntainer, BD, Franklin Lakes, NJ, USA), mixed by inverting the tube 8–10 and immediately placed on ice. Plasma was then separated by centrifugation at 1000×*g* for 10 min at room temperature. Collected plasma was visually inspected for coagulation and hemolysis, collected into 0.5 ml aliquots, and stored within 1 h of blood draw. For the saliva collection, volunteers rinsed their mouths 3 times for 30 s each with water and then dripped the saliva directly into the tube without spitting to avoid contamination with other fluids of the respiratory and gastroesophageal systems and placed on ice. Urine was collected by directly urinating into tubes and placed on ice. Immediately after collection, plasma, saliva, and urine samples were stored at − 80 °C. Sample collection, preparation and instrument run orders were randomized to minimize confounding factors.

### Ethics approval and consent to participate

The study was conducted after approval from the Institutional Review Board of the Pacific Northwest National Laboratory (PNNL IRB #2019-17) and participants signed information consent in accordance with federal regulations. The consent signed by participants of the study included permission for publishing the research findings and was captured in accordance with federal regulations.

### Sample preparation for multi-omics analysis

#### Simultaneous metabolite, protein and lipid extraction (MPLEx)

Biofluids were transferred into 2 ml Sorenson MµlTI™ SafeSeal™ tubes (Sorenson BioScience, Inc., Salt Lake City, UT, USA) with gas chromatography-mass spectrometry (GC-MS) heavy isotope internal standard mix, GC-IS (1 mg/ml in water each ^2^H_4_-malonic acid, ^2^H_4_-succinic acid, ^2^H_5_-glycine, ^2^H_4_-citric acid, ^13^C_6_-fructose, ^2^H_5_-tryptophan, ^2^H_4_-lysine, ^2^H_7_-alanine, ^2^H_35_-stearic acid, ^2^H_5_-benzoic acid, ^2^H_15_-octanoic acid): plasma − 1:1 (v:v, sample: GC-IS), urine− 4:1 (v:v, sample: GC-IS). Urine was further treated with 1 mg/ml urease at 37 °C with mild shaking (500 rpm) for 30 min and incubated on ice for 1 min. Plasma sample was also spiked with 10 μl SPLASH mix (in methanol) (Avanti Polar, Alabaster, AL, USA). Extraction was perfumed using the MPLEx protocol [16]*.* A 2:1 (v:v) chloroform/methanol mix was added to the samples to make a ratio of 8:4:3 (v:v:v) chloroform/methanol/water. Samples were then vortexed and incubated on an ice block for 5 min. The layers were separated by centrifugation at 12,000×*g* for 10 min at 4 °C. For plasma samples, 66.7 µl (from a total 500 µl extraction volume) of the lower lipid layer was transferred to a glass autosampler vial and dried in a centrifugal vacuum concentrator. The upper liquid layer and the rest of the bottom layer were transferred to another autosampler vial for metabolites and dried. For the other biofluids, the upper and lower phases were collected into separate files. The dried metabolites were capped and stored at − 20 °C for metabolomics analysis. The dried lipids had 500 µl 2:1 chloroform: methanol added, capped, and stored for lipidomics analysis. The protein pellet was washed with 1 ml of ice-cold methanol and centrifuged to the pellet, supernatant was removed and allowed to dry in a fume hood. Proteins were dissolved in 8 mol/L urea prepared in 50 mmol/L Tris-HCl, pH 8.0 and concentration was measured by BCA Protein Assay (Thermo Scientific, San Jose, CA, USA). Disulfide bonds were reduced for 1 h at 37 °C with 5 mmol/L dithiothreitol (Sigma-Aldrich, St. Louis, MO, USA) from a 500 mmol/L stock solution. Reduced cysteine residues were alkylated by adding 500 mmol/L iodoacetamide (Sigma-Aldrich, St. Louis, MO, USA) to a final concentration of 10 mmol/L and incubating in the dark at 25 °C for 45 min. Samples were diluted fourfold with 50 mmol/L Tris-HCl, pH 8.0 and digested with lysyl-C endopeptidase (FUJIFILM Wako Chemicals, Richmond, VA, USA) at 1:50 enzyme‐to‐substrate ratio at 25 °C for 2 h. The same amount of sequencing-grade modified trypsin (Promega, Madison, WI, USA) was added to the samples for 14 h incubation at 25 °C. The reaction was stopped by acidifying the samples with 100% formic acid (Sigma-Aldrich, St. Louis, MO, USA) to a final concentration of 1% formic acid, extracted in C18 SepPak cartridges (Waters, Milford, MA, USA) and dried in a centrifugal vacuum concentrator (Thermo Fisher Scientific, Carlsbad, CA, USA).

#### Metabolite extraction with methanol

Metabolites were also extracted with methanol for more comprehensive coverage. Plasma and urine samples were spiked with GC-IS as described above. Urine was also treated with urease. Eight volumes of ice-cold methanol were added, and samples were vortexed for 10 s and placed in ice blocks for 10 min and vortexed for 3 min. Centrifuged at 15,000×*g* at 4 °C for 10 min and the supernatant was transferred to autosampler vials and dried in a centrifugal vacuum concentrator. Dried metabolites were capped and stored at − 20 °C for further metabolomics analysis.

#### Plasma sample preparation for isobaric labeled proteomic analysis

Plasma abundant proteins were depleted using a Multiple Affinity Removal System (MARS) column (Hu-14 4.6 × 100 mm, Agilent Technologies, Santa Clara, CA, USA) coupled with a 1200 series HPLC (Agilent Technologies, Santa Clara, CA, USA). A total of 40 μl of plasma was diluted eightfold with Agilent buffer A and filtered with a 0.22 µm centrifugal filter. Samples were loaded onto MARS column for 27 min LC gradient (18 min sample load at 0.125 ml/min, then washed for 2 min at 1 ml/min with Agilent buffer A, and high abundant proteins eluted for 7 min at 1 ml/min Agilent buffer B). Unbound fractions, containing low- and mid-abundant proteins from the same participant were pooled, then concentrated and had their buffer exchanged to 50 mmol/L Tris-HCl, pH 8.0 using 3-kD molecular mass cutoff Amicon centrifugal filters (Millipore, Burlington, MA, USA) following the manufacturer’s instructions. Proteins were digested as described above and labeled with 11-plex tandem mass tags (TMT, Thermo Fisher Scientific Carlsbad, CA, USA) according to the manufacturer’s recommendations. One of the TMT channels was loaded with a pooled peptide mixture from all the samples, which serves as a reference to normalize across different sets of samples. Labeled peptides were fractionated into 96 fractions by high pH reversed-phase chromatography and concatenated into 24 fractions, as previously described [17, 18].

### Proteomics analysis

Peptides were analyzed by liquid chromatography-tandem mass spectrometry (LC-MS/MS) using a nanoAquity UPLC^®^ system (Waters Corporation, Milford, MA, USA) connected to a Q-Exactive mass spectrometer (Thermo Scientific, San Jose, CA, USA) as described in detail elsewhere [19]. Data from the TMT-labeled experiment were processed with Decon2LS software combined with DTA Refinery (version 2, Pacific Northwest National Laboratory, Richland, WA, USA) [20, 21] for mass recalibration and peak list extraction. Peptides were identified with MSGF+ [22] by searching against the human version of the SwissProt database downloaded from Uniprot Knowledgebase on February 22, 2019. The searching parameters consisted of 1) parent ion mass tolerance of ± 6 ppm, 2) tryptic digestion in at least one of the termini with 2 missed cleavages allowed, 3) cysteine carbamidomethylation (+ 57.0215 Da) and *N*-terminal/lysine TMT labeling (+ 229.1629 Da) derivatization as static modifications, and 4) following variable modifications: oxidation (+ 15.9949 Da) on methionine, cysteine, tyrosine and tryptophan; dioxidation (+ 31.9898 Da) on cysteine; and deamidation/deamination (+ 0.98402 Da) on asparagine, glutamine and arginine residues. Data were filtered at spectral-peptide match (MSGF probability ≤ 1 × 10^−9^), peptide (MSGF probability ≤ 7 × 10^−11^) and protein (MSGF probability ≤ 2 × 10^−12^) levels, resulting in < 1% false-discovery rate in each of the levels. TMT reporter ion intensities were extracted with MASIC [23] (version 1, Pacific Northwest National Laboratory, Richland, WA, USA), and the intensities of multiple MS/MS spectra from the same peptide were summed together to remove redundancy.

Label-free proteomics data were processed with MaxQuant (version 1.6.5.0, Max Planck Institute, Planegg, Germany) [24] by searching tandem mass spectra against the human proteome database downloaded from Uniprot Knowledgebase (https://www.uniprot.org/) on September 23, 2019. Searching parameters considered trypsin cleavage in both peptide termini, methionine oxidation and protein N-terminal acetylation as variable modifications and cysteine carbamidomethylation as fixed modification. Mass tolerance of parentions was set to 20 and 4.5 ppm for prior and after mass recalibration, respectively. The remaining parameters were set as the software default options. Resulting identifications were filtered with a ≤ 1% false-discovery rate in both peptide-spectrum match and protein levels. Label-free quantification and intensity-based absolute quantification methods were used for the MaxQuant analysis. For this analysis, the match between runs option was enabled to decrease missing values.

The saliva microbiome analysis was processed with Decon2LS software combined with mzRefinery (version 2, Pacific Northwest National Laboratory, Richland, WA, USA) [25] for mass recalibration and peak list extraction. Peptides were identified with MSGF + using the human SwissProt database combined with the Human Oral Microbiome Database (downloaded from https://www.homd.org/ on February 17, 2020). The searching parameters consisted of 1) parent ion mass tolerance of ± 20 ppm, 2) tryptic digestion of both termini with 2 missed cleavages allowed, 3) cysteine carbamidomethylation (+ 57.0215 Da) as invariable modification and 4) methionine oxidation (+ 15.9949 Da) as variable modification. Data were filtered at spectral-peptide match (MSGF probability ≤ 1.0 × 10^−9^), peptide (MSGF probability ≤ 1 × 10^−11^) and protein (MSGF probability ≤ 1 × 10^−12^) levels, resulting in < 1% false-discovery rate in each of the levels. The results led to the identification of proteins from 142 bacterial genera. For the quantitative analysis, we appended the top strain of each of the 142 bacterial genera to the human SwissProt database (both downloaded from https://www.uniprot.org/ on April 5, 2020) and reanalyzed the data with MaxQuant v.1.6.14, which can identify and quantify proteins but performs better in smaller sequence databases [26]. The same parameters for the label-free proteomics analysis were used to process the data.

### Metabolomic analysis

Metabolites were derivatized with N-methyl-N-(trimethylsilyl)trifluoroacetamide (MSTFA) (Sigma-Aldrich, Saint Louis, MO, USA) and trimethylchlorosilane (TMCS) (Sigma-Aldrich, Saint Louis, MO, USA) and analyzed on Agilent GC 7890A using a HP-5MS column (30 m × 0.25 mm × 0.25 μm; Agilent Technologies, Santa Clara, CA, USA) coupled with a single quadrupole MSD 5975C (Agilent Technologies, Santa Clara, CA, USA) as previously described [27]. Fatty acid methyl ester standard mix (C8-28) (Sigma-Aldrich, Saint Louis, MO, USA) was analyzed in parallel as standard for retention time calibration. Collected data were calibrated and deconvoluted using Metabolite Detector (version 2, Technical University, Braunschweig, Germany) [28]. Identification of molecules was done by matching against the FiehnLib library [29] with additional in-house entries and the NIST17/Wiley 11 GC-MS spectral databases.

### Lipidomic analysis

Lipids were subjected to LC-MS/MS analysis on orbitrap mass spectrometry (Velos Orbitrap, Thermo Fisher Scientific, San Jose, CA, USA) as previously described [30]. Lipid species were identified using LIQUID (version 1, Pacific Northwest National Laboratory, Richland, WA, USA) [30] and identifications were manually validated based on the MS/MS spectra (diagnostic and corresponding acyl chain fragments), the precursor isotopic profile, extracted ion chromatogram, mass measurement accuracy and elution time. Quantification was performed with MZmine (version 2, VTT Technical Research Centre of Finland) [31]. All LC-MS/MS data were aligned and gap-filled to the identified lipid-based observed m/z and retention time. Aligned features were manually verified and peak apex intensity values were exported for statistical analysis.

### Dermcidin ELISA assay

Dermcidin was quantified using an ELISA kit (catalog number MBS2704747, MyBioSource, San Diego, CA, USA). One-hundred microliters of plasma or saliva were plated onto 96-well plates and incubated for 1 h at 37 °C, then add Detection Reagent A and incubate for another hour at 37 °C. After this period, plates were washed 3 times with 350 μl of wash buffer before incubation with appropriate Detection Reagent B for 30 min at 37 °C. Plates were washed 5 times with 350 μl of wash buffer and incubated with 90 μl of Substrate Solution for 20 min at 4 °C. The reaction was quenched with 50 μl of Stop Solution and measured at 450 nm in a plate spectrophotometer.

### Urea quantification assay

Urine urea concentration was determined with a urease-based kit (Sigma-Aldrich, Saint Louis, MO, USA) following manufacturer-provided protocol. Fifty microliters of reaction mix containing Urea assay buffer, Peroxidase substrate, Enzyme mix, Developer and Converting Enzyme, was mixed with 50 μl of 1500-fold diluted urine and incubated for 60 min at 37 °C. The reaction was measured at 570 nm in a plate spectrophotometer.

### Systematic literature review

The literature search was done between February 26 and March 16, 2021, by querying PubMed for publications (see Table 1 for keywords). The groups of workers that were included in this search were firefighters, soldiers, marathon runners and soccer players. Only studies that followed up individuals for respiratory infections after physical activity were included in the analysis.

**Table 1:** Systematic literature review on respiratory infection incidence upon intense physical demanding tasks

Keywords used for PubMed search	Number of papers	Qualified papers	Reference	Study design	Outcomes of the study
Firefighter respiratory infection OR firefighter exhaustion infection OR firefighter intense physical activity infection OR firefighter training infection	79	1	[65]	The study monitored 58 wildland firefighters for respiratory symptoms markers during preseason, postfire, and postseason	Upper and lower respiratory symptom scores were higher postfire compared to preseason or postseason
Soldier training intensity respiratory infection	9	1	[66]	A cohort of 21 soldiers underwent to 3 weeks of training followed by a 5 days combat course with energy restriction, sleep deprivation and psychological stress. The training consisted of swimming, walking and running in rough terrain. In trails, individuals carried backpacks of (11 ± 1.2) kg	A total of 30 upper respiratory infection episodes were recorded. These episodes were similarly distributed during training and combat course but reduced drastically after 2 days of recovery
Marathon respiratory infection OR marathon runner infection	118	4	[67]	Followed up 208 runners of the 2010 London Marathon for 15 post-run via questionnaire. The results were compared to a control group of 128 football players, who did not run the marathon	47% of runners reported respiratory symptoms versus 19% of non-runners. Part of the symptoms can also be attributed to allergies as well
			[68]	The study followed up 141 participants of the 1982 race from Pretoria to Johannesburg (56 km) for 2 weeks after the race via questionnaire. The control was a group of 125 individuals that lived with the participants	33.3% of the runners reported respiratory infections versus 15.3% of the control group
			[70]	The study followed up 34 participants (21 races and 13 controls) of the 18th Oita International Wheelchair Marathon for 1 month prior the race and 2 weeks after race via questionnaire	No significant changes were found
			[69]	The study followed up 2311 participants (1828 who completed the race and 134 controls who chose not to run, the remaining failed to complete the race and were excluded from the study) of the 1987 Los Angeles Marathon for a week before and a week after the run via questionnaire	12.9% of runners had respiratory infections after the marathon compared to 2.2% of the control group
Physical demand respiratory infection	209	1	[71]	The study monitored 34 boys during 12 weeks preparatory training phase, 7 weeks competition and 2 weeks post-season	Upper respiratory symptom scores were significantly reduced in the post-season period compared to training and competition phases

### Statistical analysis

Statistical analyses were conducted in R version 3.6 using the pmartR [32] and stats packages. Missing values were converted to “NA” and data were Log_2_ transformed. TMT-labeled proteomics data were normalized to the reference pool. Peptides shared by multiple proteins were excluded. Biomolecules appearing only once across all samples were excluded. A robust Mahalanobis distance based on peptide abundance vectors (rMd-PAV) was calculated to identify potential sample outliers in the data [33]. Sample outliers were confirmed using visual inspection of correlation heatmaps and principal component analysis (PCA) plots. The metabolomics and lipidomics datasets were normalized via global median centering. Proteomics datasets were normalized by the statistical procedure for the analyses of peptide abundance normalization strategies (SPANS) [34] at the peptide level, and rolled up with R-rollup. Statistical comparisons of biomolecule abundances were performed using a paired Student’s *t*-test and considered significant with a *P*-value ≤ 0.05 without further corrections.

### Functional-enrichment analysis

Differentially abundant proteins (Student’s *t*-test *P*-value ≤ 0.05) were submitted for functional-enrichment analysis using DAVID (version 6.8, Frederick National Laboratory for Cancer Research, Frederick, MD, USA) [35] and the whole set of human predicted genes was used as the background. Only the pathways containing KEGG annotation were considered in the analysis. Graphs of pathways overrepresented with differentially abundant proteins were plotted with Minitab (version 19.2020.1, Minitab LLC, State College, PA, USA). Lipid ontology and enrichment analysis were done using Lipid Mini-On (version 1, Pacific Northwest National Laboratory, Richland, WA, USA) [36].

## Results

Blood plasma, urine, and saliva were collected from wildland firefighters before and after an exercise, which consisted of hiking for 45 min, at a strenuous pace, in hilly terrain, while wearing full wildland firefighter gear (between 9 and 20 kg). Exercise sessions occurred at the start of the fire season, in late June 2019, during onboard training. The local temperature was 20 °C, while the air humidity was 78% with 6 km/h winds. From the initial 13 male firefighters, two participants were excluded, one was unable to finish the exercise regimen and a second study participant exceeded the post-exercise biospecimen collection time threshold. Participants that concluded the course were on average (25 ± 3) years old and weighed on average (80.1 ± 13.5) kg before exercise, and lost, on average, (1.8 ± 0.2) kg (2.2% of the initial weight) during exercise. The participants’ body mass index reduced from (26.6 ± 3.3) kg/m^2^ pre-exercise to (26.0 ± 3.3) kg/m^2^ post-exercise. Each biofluid was submitted for comprehensive proteomics, lipidomics and metabolomics analyses. Combining all the analyses 3835 proteins, 730 lipids and 182 metabolites were identified and quantified (Additional file 1: Tables S1–S3). The different omics measurements were then integrated to provide a global view of physiological and biochemical pathways regulated during the exercise session that is released into different biofluids.

### Multi-omics signatures of intense exercise in plasma

**Fig. 1:**
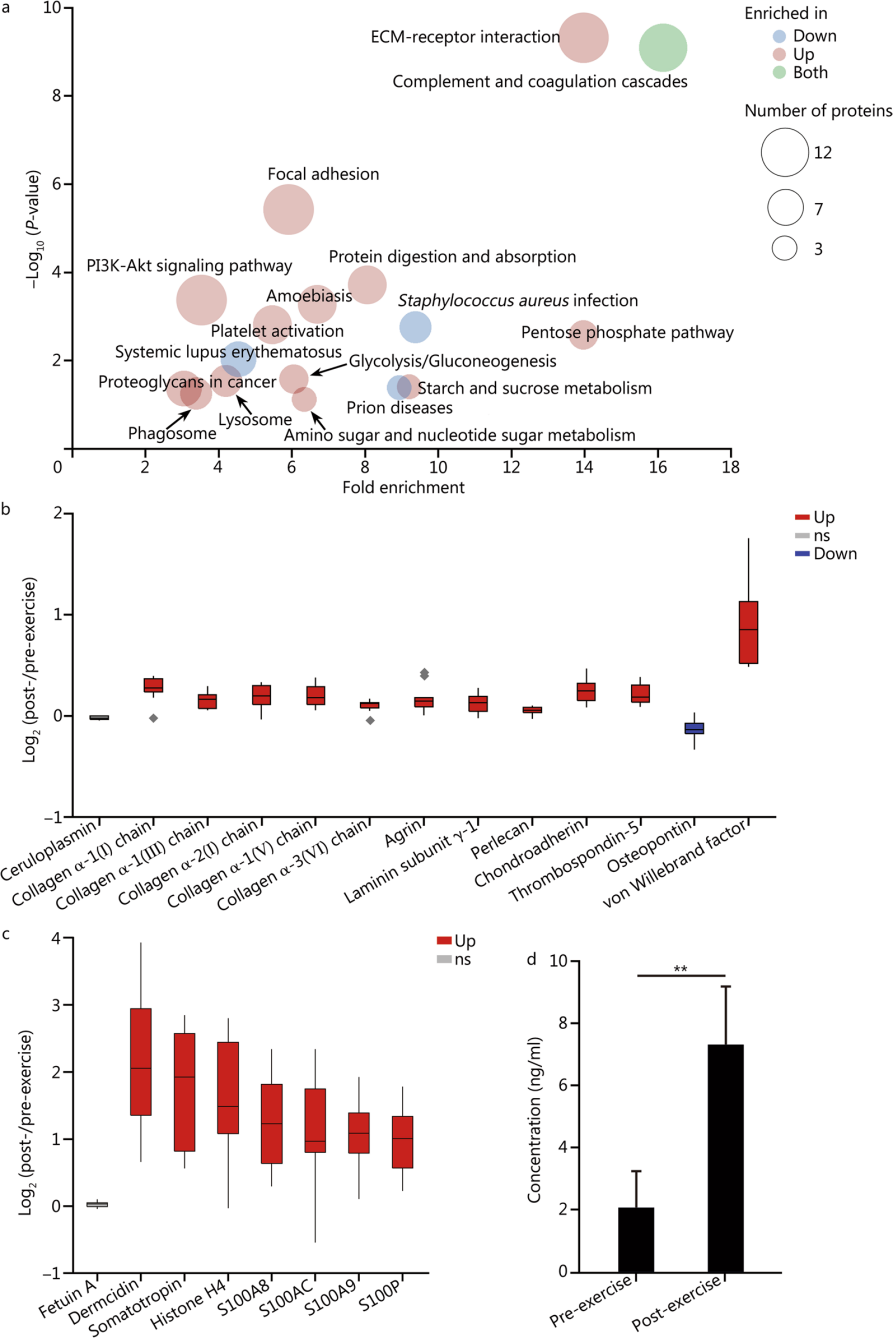
Comparative multi-omics analysis of blood plasma prior and post exercise. **a** Functional-enrichment analysis of proteins differentially abundant (Student’s *t*-test *P* ≤ 0.05) after the exercise session. The enrichment analysis was done with DAVID and the graph is plotted in function of the fold enrichment versus Fisher’s exact test *P*-values. The colors represent if the pathways were overrepresented in up-regulated or down-regulated proteins, while the circle sizes represent the number of regulated proteins in each pathway. **b** Boxplot of abundance ratios of extracellular matrix (ECM) proteins comparing pre- and post-exercise sessions. Black diamonds represent outlying data points. **c** Boxplot of abundance ratios of regeneration factors comparing pre- and post-exercise sessions. **d** ELISA analysis of plasma dermcidin levels prior and after the exercise session. ^**^P ≤ 0.01 (Student’s *t*-test). Down significantly down-regulated molecule, Up significantly up-regulated molecule, ns non-significant

The plasma proteomics analysis resulted in the identification and quantification of 1510 proteins. Out of the 1510 identified proteins, 142 significantly (Student’s* t*-test *P* ≤ 0.05, 107 up-regulated and 35 down-regulated) changed across the pre- and post-exercise plasma samples (Additional file 1: Table S4). The paired metabolomics analysis of the same samples led to the identification of 91 metabolites, 29 up-regulated and 35 down-regulated (Additional file 1: Table S5). A lipidomics analysis resulted in the identification of 391 lipid species, 149 of which were up-regulated and 77 of which were down-regulated (Additional file 1: Table S6). A functional-enrichment analysis of the proteomics data shows that pathways related to *Staphylococcus aureus* infection, systemic lupus erythematosus and prion diseases were down-regulated post-exercise (Fig. 1a), possibly indicating immune modulation. Complement and coagulation cascades had proteins simultaneously down-regulated and up-regulated post-exercise, while the coagulation proteins were up-regulated, and the complement proteins were down-regulated (Fig. 1a and Additional file 1: Table S4). Enrichment of pathways such as ECM-receptor interaction, focal adhesion, and proteoglycans in cancer (Fig. 1a) showed a general increase in extracellular matrix (ECM) proteins. Except for osteopontin, all the other 11 proteins of the ECM-receptor interaction pathway were up-regulated post-exercise (Fig. 1b). As a loading control, the abundant plasma protein, ceruloplasmin, was unaffected by the exercise session (Fig. 1b). The release of ECM proteins into the blood might be due to tissue damage.

We also investigated the proteins with the most up-regulation following the exercise regimen. Dermcidin, an antimicrobial peptide and regulator of glucose metabolism, was the most up-regulated protein with a 4.4-fold increase in abundance in the proteomics analysis (Fig. 1c). A validation experiment with ELISA showed a 3.5-fold increase in dermcidin after exercise (Fig. 1d). Subsequent significant proteins with up-regulation were all growth and tissue regeneration factors, such as somatotropin and several S100 proteins (Fig. 1c). Histone H4, a major structural protein of cellular chromatins, was up-regulated 3.3-fold (Fig. 1c). The presence of chromatin proteins in the plasma is surprising to some extent but could be caused by cell lysis. To check this possibility, we looked at the other histones. Histone H2A type 2-C abundance was below the limit of quantification (Additional file 1: Table S1), while the other core histones that are equally as abundant as H4 in cells, such as H2A, H2B and H3, were not detected. We also found that the two peptides of histone H4 detected in our analysis were from its C-terminal region. The 5 amino acid residues of histone H4 C-terminus correspond to the osteogenic growth peptide, a tissue regeneration factor. Therefore, the identified peptides from histone H4 are probably secreted osteogenic growth peptides rather than leakage of lysed cells. The pathways related to immune response (Fig. 1a) could also be contributing to tissue repair. Overall, these signatures suggest an acute response to the tissue damage toward regeneration.

**Fig. 2:**
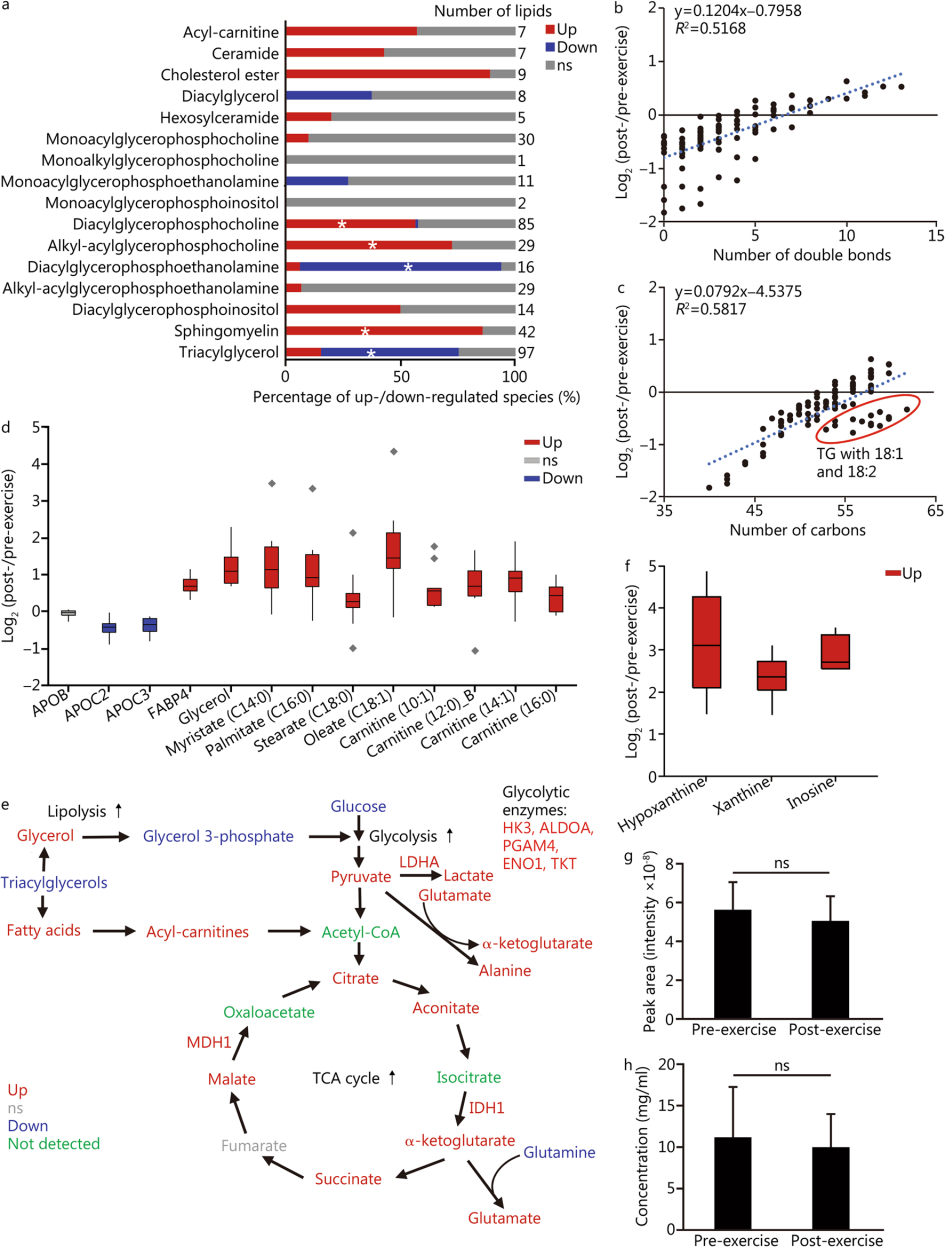
Metabolic signatures of the exercise session in the blood plasma. **a** Plasma lipidomics profile comparing prior and after the exercise session. The bar graph shows the percentage up and down-regulated species in each lipid class. The asterisks represent classes of lipids that are significantly enriched (Fisher’s exact test *P* ≤ 0.05) with differential abundant species, as determined using Lipid MiniOn. **b** Relationship between the total number of double bonds in triacylglycerol species and fold change comparing post- versus pre-exercise. **c** Relationship between the total number of carbons in fatty acids of triacylglycerol species and fold change comparing post- versus pre-exercise. **d** Boxplot of abundance ratios of lipid metabolism molecules comparing pre- and post-exercise sessions. Diamonds represent outlying data points. **e** Levels of molecules from the central carbon metabolism in plasma comparing pre- and post-exercise sessions. **f** Boxplot of abundance ratios of ATP catabolites in plasma comparing pre- and post-exercise sessions. **g** Relative quantification of the plasma urea levels using the GC–MS-based metabolomics data. **h** Quantification of the urine urea concentrations using a colorimetric assay. ALDOA aldolase A, Down significantly (Student’s *t*-test *P* ≤ 0.05) down-regulated molecule, ENO1 enolase 1, HK3 hexokinase 3, IDH1 isocitrate dehydrogenase 1, LDHA lactate dehydrogenase, ns non-significant, PGAM4 phosphoglycerate mutase family member 4, Up significantly up-regulated molecule, ns non-significant, TG triacylglycerol, TKT transketolase

The lipidomics analysis showed differential abundance of multiple lipid classes. The sphingolipids [sphingomyelin (SM), ceramides and hexosylceramides], largely driven by SM, have several up-regulated lipid species (Fig. 2a). Among the phospholipids, diacylglycerophosphoethanolamines (PEs) and monoacylglycerophosphoethanolamines (LPEs) were down-regulated, whereas diacylglycerophosphocholines (PCs), acylalkylglycerophosphocholines (PCOs) and diacylglycerophosphoinositols (PIs) were up-regulated (Fig. 2a). In terms of energy storage lipids, a decrease of several species of diacylglycerol (DG) and triacylglycerol (TG) (Fig. 2a). Conversely, TGs containing very long, polyunsaturated fatty acid C22:6 were statistically enriched among the lipids with increased abundance post-exercise (Additional file 1: Table S5). To better understand this process, we sorted TG species based on their fold change against double bonds or total carbon length. TGs with fewer double bonds had the highest decrease, whereas the more unsaturated ones were up-regulated (Fig. 2b). TGs with a smaller carbon number also had the highest decrease (Fig. 2b), suggesting a preference for the degradation of TGs with short saturated fatty acids. One exception to this rule is the TGs with C18:1 and C18:2, which are degraded faster than TGs with C18:0 (Fig. 2b). In agreement with this observation, the level of oleate (C18:1) was increased by 66% (*P* = 0.001) in plasma compared to non-significant changes of the stearate (C18:0) level (*P* = 0.17) (Fig. 2b). The lipolysis activator apolipoprotein C3 (found on triglyceride-rich lipoproteins) was up-regulated by 28% (*P* = 0.0002), while the lipolysis marker fatty acid-binding protein 4 was increased by 65% (*P* < 0.001), indicating an increase in lipolysis (Fig. 2c). Consistently, the lipolysis products glycerol (+ 55%) and fatty acids myristate (C14:0): + 57% (*P* = 0.001), and palmitate (C16:0): + 51% (*P* = 0.004) were also increased post-exercise (Fig. 2d). The increase in plasma fatty acid levels was accompanied by increase in the levels of 10:1 (+ 53%, *P* = 0.003), 12:0 (+ 59%, *P* = 0.01), 14:1 (+ 74%, *P* = 0.001) and 16:0 (+ 31%, *P* = 0.007) acyl-carnitines (Fig. 2c), which are conjugated molecules that facilitate the transport of fatty acids to the mitochondria for beta oxidation.

Downstream analysis of the metabolites from central carbon metabolism showed that glucose had only a 7% reduction (*P* = 0.02) in abundance post-exercise (Additional file 1: Table S6). This small change could be due to the homeostatic mechanisms to maintain the blood glucose levels as we observed changes in D-gluconic acid and in other sugars along with isomerases and kinases (Additional file 1: Tables S4 and S6). An increase in the levels of the glycolysis products, pyruvate (+ 51%, *P* < 0.001) and lactate (+ 34%, *P* = 0.02) (Fig. 2e), was also observed, which agrees with the proteomics data showing enrichment in glycolysis/gluconeogenesis (Fig. 1a). In addition, we observed 5 out of the 10 glycolytic enzymes being up-regulated: hexokinase HK3, aldolase ALDOA, phosphoglycerate mutase PGAM4, enolase ENO1 and transketolase TKT (Fig. 2e). A similar increase was observed in the levels of 5 out of 7 TCA cycle intermediates and 2 enzymes in the plasma samples (Fig. 2e). We also aimed to look for the ATP levels, but neither ATP nor its catabolites were detected by the automated GC-MS analysis. After manual inspection, we found that the increase of glycolysis and TCA cycle metabolites was accompanied by elevated levels of ATP catabolites in plasma: hypoxanthine (+ 11.6-fold, *P* < 0.001), xanthine (+ 5.2-fold, *P* < 0.001) and inosine (+ 7.3-fold, *P* = 0.001) (Fig. 2f). In terms of amino acids, glutamine, and arginine (detected as ornithine) were reduced by 17% (*P* = 0.003) and 25% (*P* < 0.001), respectively (Additional file 1: Table S3). Conversely, alanine and glutamate levels were increased by 23% (*P* = 0.01) and 66% (*P* = 0.001) (Additional file 1: Table S6), while all other detected amino acids had minor or insignificant changes in abundance. To further investigate if amino acids are used as an energy source, we analyzed the levels of their degradation product, urea, in plasma and urine. The levels of urea in plasma and urine were measured by GC-MS and enzymatic assay, respectively, and neither of them showed differences after exercise (Fig. 2g, h), supporting that amino acid catabolism was not an important source of energy during this exercise session.

Overall, in plasma we observed an increase in ECM (tissue damage markers), immunomodulation and regeneration proteins, suggesting that the impact of exercise causes tissue damage and that the repair response starts immediately. A strong change in lipid metabolism, glycolysis and TCA cycle was observed, probably to support the high energy demands of the body.

### Multi-omics signatures of intense exercise in urine

**Fig. 3:**
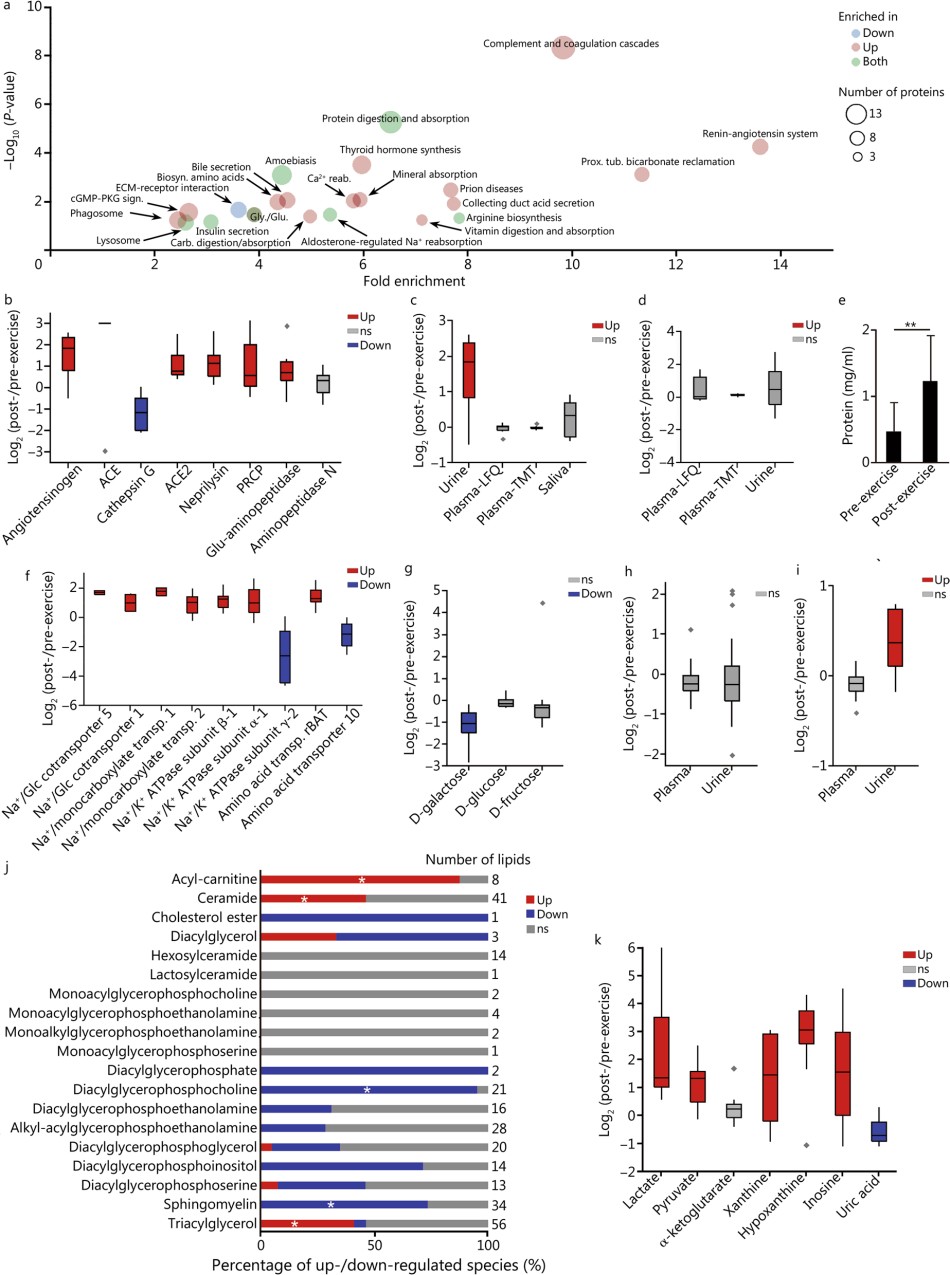
Comparative multi-omics analysis of urine prior and post exercise. **a** Functional-enrichment analysis of proteins differentially abundant (Student’s *t*-test *P* ≤ 0.05) after the exercise session. The enrichment analysis was done with DAVID and the graph is plotted in function of the fold enrichment versus Fisher’s exact test *P*. The colors represent if the pathways were overrepresented in up-regulated or down-regulated proteins, while the circle sizes represent the number of regulated proteins in each pathway. **b** Boxplot of abundance ratios of renin-angiotensin system proteins comparing pre- and post-exercise sessions. **c** Boxplot of abundance ratios of angiotensinogen in different body fluids comparing pre- and post-exercise sessions. **d** Boxplot of abundance ratios of cystatin C in different body fluids comparing pre- and post-exercise sessions. **e** Protein content in the urine prior to post exercise. ^**^P < 0.01 (Student’s *t*-test). **f** Boxplot of abundance ratios of transporters comparing pre- and post-exercise sessions. **g** Boxplot of abundance ratios of urine sugar levels comparing pre- and post-exercise sessions. **h** Boxplot of abundance ratios of valine pre- and post-exercise session comparing plasma and urine. **i** Boxplot of abundance ratios of cysteine pre- and post-exercise session comparing plasma and urine. **j** Urine lipidomics profile comparing prior and after the exercise session. The bar graph shows the percentage up- and down-regulated species in each lipid class. The stars represent classes of lipids that are significantly enriched (Fisher’s exact test *P* ≤ 0.05) with differential abundant species, as determined using Lipid MiniOn. **k** Boxplot of abundance ratios of metabolites comparing pre- and post-exercise sessions. Diamonds represent outlying data. ACE angiotensin converting enzyme, ACE2 angiotensin converting enzyme 2, Biosyn. biosynthesis, Down significantly down-regulated molecule, ns non-significant, PRCP prolylcarboxypeptidase, Prox. tub. proximal tubule, reab. reabsorption, sign. signaling, transp. transporter, Up significantly up-regulated molecule

The multi-omics analysis of the urine samples resulted in the identification and quantification of 1711 proteins, 105 metabolites and 279 lipids (Additional file 1: Tables S7–S9). Out of the molecules, 291 proteins (197 up-regulated and 94 down-regulated), 37 metabolites (14 up-regulated and 23 down-regulated) and 139 lipids (52 up-regulated and 87 down-regulated) were differentially abundant between pre- and post-exercise urine samples. The functional-enrichment analysis showed a strong trend in the regulation of secretion and reabsorption pathways (Fig. 3a). The renin-angiotensin system pathway, which controls body fluid balance and blood pressure, was enriched 13.6-fold among the post-exercise-regulated proteins (Fig. 3a). In addition, we found the level of angiotensinogen in urine was increased 2.9-fold after the exercise session, and the levels of its converting enzymes were also consistently increased: angiotensin-converting enzyme (ACE) (detected in 6 samples post-exercise and only 1 before exercise), angiotensin-converting enzyme 2 (ACE2) (+ 2.1-fold, *P* = 0.001), Lysosomal Pro-X carboxypeptidase (+ 2.0-fold, *P* = 0.02), neprilysin (+ 2.2-fold, *P* = 0.0007) and glutamyl aminopeptidase (+ 1.8-fold, *P* = 0.02). The level of cathepsin G was reduced by 2.2-fold (*P* = 0.007) and the level of aminopeptidase N was not significantly different (*P* = 0.25) (Fig. 3b). We observed increases in angiotensin in urine, but not blood or saliva (Fig. 3c). As the renin-angiotensin pathway increases vasoconstriction, we investigate if it would alter the filtration rates of the kidney, we look at the levels of cystatin C. Cystatin C is a continuously expressed freely filtered small protein used clinically as a marker of glomerular filtration rate. Since cystatin is completely catabolized in the proximal tubules of the kidney, plasma levels of cystatin C are used as clinical biomarker for filtration rates. We found that the level of cystatin C in plasma was similar pre- versus post-exercise session (Fig. 3d). Similarly, the plasma level of creatinine, another indicator of glomerular filtration rate, was also not significantly affected by the exercise session (Additional file 1: Table S3). This indicates that glomerular filtration rates are maintained at an approximately constant level during exercise. Conversely, we found an increase in total protein concentration of the urine post-exercise (*P* = 0.0057) (Fig. 3e), which might indicate an increase in water reabsorption by the renin-angiotensin system to aid in water body homeostasis (i.e., combat water loss due to perspiration).

We next investigated the effect of the renin-angiotensin system regulation in more detail by analyzing the abundance of ion and metabolite transporters. We found a significant change in the abundance of 12 transporters, including 10 up-regulated and 2 down-regulated after exercise (Additional file 1: Table S4). The abundance of sodium/glucose cotransporters 1 and 5, which are involved in sugar reabsorption, were up-regulated by 2.0- (*P* = 0.05) and 3.3-fold (*P* = 0.0002), respectively (Fig. 3f). Concomitantly, the level of galactose was reduced by 2.3-fold (*P* = 0.001), while the levels of glucose and fructose remained similar (Fig. 3g). We also observed an increase in abundance of the sodium/potassium-transporting ATPase (ATP1B1: 2.3-fold, *P* < 0.001 and ATP1A1: 2.1-fold, *P* = 0.006), while the regulatory subunit γ-2 was down-regulated by 5.9-fold (*P* = 0.009) (Fig. 3g). In addition, the sodium-coupled monocarboxylate transporters 1 and 2 were up-regulated by 3.4- (*P* = 0.008) and 1.8-fold (*P* = 0.005) (Fig. 3f), respectively, suggesting a reabsorption of ions and consequently, water. The neutral and basic amino acid reabsorption transport protein rBAT (SLC3A1) was increased by 2.6-fold (*P* < 0.001) after exercise. Conversely, the level of neutral amino acid transporter 10 (SLC38A10) was reduced by 1.1-fold (*P* = 0.01) (Fig. 3f). Most of the amino acids had similar post-/pre-exercise fold changes comparing plasma with urine (Fig. 3h and Additional file 1: Table S6). However, there were amino acids, such as cysteine, which responded differently in plasma than in urine. Cysteine abundance increased 29% (*P* = 0.003) post-/pre-exercise in urine while plasma levels remained similar (Fig. 3i). These data support differential reabsorption of amino acids during exercise.

In terms of energy-related metabolites, the lipidomics analysis of urine showed a strikingly different profile compared with the plasma samples. Despite similarly higher levels of acyl-carnitines and ceramides in urine and plasma, the levels of TG, SM, PC, PCO and PI were divergent in these biofluids (Figs. 2a and 3j). TG levels were higher in urine, while the levels of SM, PC, PCO and PI were lower (Fig. 3j). These differences in lipid profiles between plasma and urine might be due to filtration, reabsorption, and differences in energetic demands locally in the kidneys. The glycolysis products, lactate and pyruvate, were increased by 4.7- (*P* = 0.001) and 2.2-fold (*P* = 0.0004), respectively (Fig. 3k). On the other hand, the levels of most the TCA cycle intermediates (Additional file 1: Table S3) were similar before and after exercise, like that of α-ketoglutarate (Fig. 3k). The levels of the ATP catabolites, xanthine, hypoxanthine, and inosine, were increased after exercise, while the level of uric acid was decreased at the same time (Fig. 3k). Collectively, the observed increases in urinary catabolites are consistent with a urinary molecular signature of an increase of the energy utilization.

Our data showed a simultaneous and consistent molecular signature of energy utilization and catabolite excretion in the plasma and urine. We also observed increases in the renin-angiotensin system and nutrient re-absorption system proteins, consistent with an adaption to maintain fluid balance and meet the increased energy demands of exercise.

### Multi-omics signatures of intense exercise in saliva

**Fig. 4:**
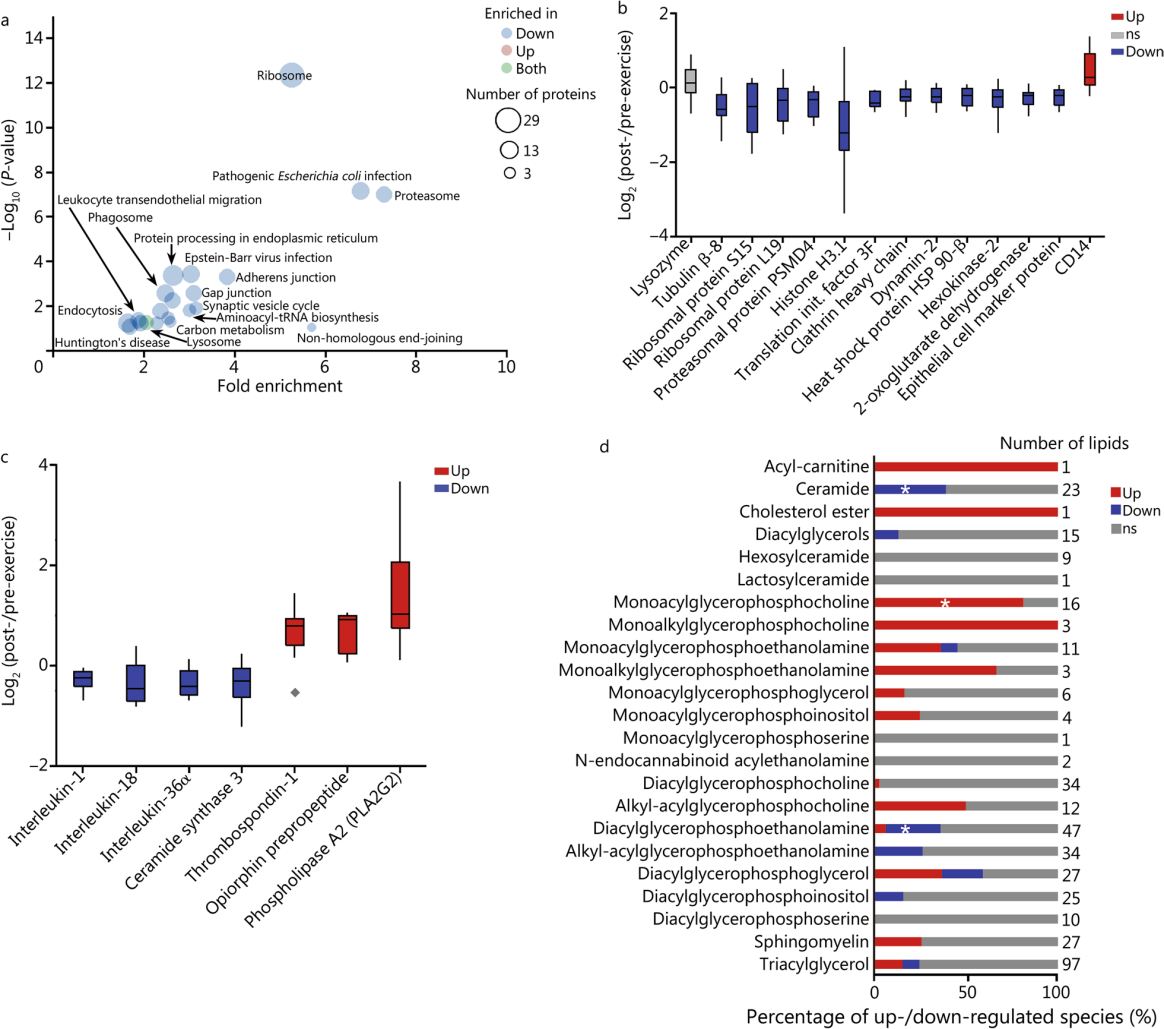
Comparative multi-omics analysis of saliva prior and post exercise. **a** Functional-enrichment analysis of proteins differentially abundant (*Student’s t*-test *P* ≤ 0.05) after the exercise session. The enrichment analysis was done with DAVID and the graph is plotted in function of the fold enrichment versus Fisher’s exact test *P*-values. The colors represent if the pathways were overrepresented in up-regulated or down-regulated proteins, while the circle sizes represent the number of regulated proteins in each pathway. **b** Boxplot of abundance ratios of abundant intracellular proteins comparing pre- and post-exercise sessions. Diamonds represent outlying data points. **c** Boxplot of abundance ratios of inflammation and fluid balance comparing pre- and post-exercise sessions. **d** Saliva lipidomics profile comparing prior and after the exercise session. The bar graph shows the percentage of up and down-regulated (Student’s *t*-test *P* ≤ 0.05) species in each lipid class. The stars represent classes of lipids that are significantly enriched (Fisher’s exact test *P* ≤ 0.05) with differential abundant species, as determined using Lipid MiniOn. Down down-regulated molecule, init. initiation, Up up-regulated molecule, ns non-significant

The multi-omic analysis of the saliva samples resulted in the identification and quantification of 2339 human proteins, 93 metabolites and 410 lipids (Additional file 1: Tables S10–S12). Of these molecules, 487 proteins (92 up-regulated and 395 down-regulated), 7 metabolites (4 up-regulated and 3 down-regulated) and 122 lipids (68 up-regulated and 54 down-regulated) were significantly regulated in response to the exercise regimen. A functional-enrichment analysis with DAVID showed 23 pathways to be overrepresented in proteins regulated by the exercise session. We found an overrepresentation of down-regulated proteins from highly abundant intracellular pathways, such as ribosome, proteasome, carbon metabolism, gap junctions and aminoacyl-tRNA synthesis (Fig. 4a, b). In contrast, the level of lysozyme, which is produced by the submaxillary gland and directly secreted into saliva, was not significantly affected by exercise (Fig. 4b). The consistent decrease in proteins from several abundant intracellular pathways post- versus pre-exercise may suggest a reduction of the cell numbers in saliva. A 17% (*P* = 0.01) reduction in the epithelial cell marker protein 1 (also known as 14-3-3 sigma) was observed while the myeloid cell marker CD14 level increased by 40% (*P* = 0.02) (Fig. 4b), suggesting that the decrease in abundant cellular proteins may be due to a reduction of epithelial cell shedding into saliva.

We also observed a decrease in the levels of the pro-inflammatory cytokines IL-36α, IL-18, and IL-1 (Fig. 4c). This was accompanied by a decrease in the level of ceramides (Fig. 4d), which are also pro-inflammatory molecules. Consistent with this observation, ceramide synthase 3 was also reduced after exercise (Fig. 4c). We also observed an increase in levels of anti- or post-inflammatory lipid species, such as monoalkylglycerophosphocholines (also known as lyso-PAF) and TGs (Fig. 4d). These data show a coordinated reduction of inflammation post-exercise, which is further supported by a reduction in the leukocyte trans-endothelial migration pathway (Fig. 4a). One possibility is that this reduction in inflammation facilitates gas exchange since airway inflammation may induce bronchoconstriction or obstructive airflow patterns. However, salivary biomarker composition may not be reflective of that found within nasal secretions, lower respiratory tract secretions or alveolar lung fluid. Thrombospondim-1, an inducer of the pulmonary vasoconstriction, was up-regulated by 57% (*P* = 0.04) after exercise, while opiorphin prepropeptide, a vasodilator of the peripheral tissue, was up-regulated by 60% (*P* = 0.02) (Fig. 4c. We also observed an up-regulation of secreted phospholipase A2 (PLA2G2) (Fig. 4c and its products monoacylglycerophosphocholines (also known as lysoPCs) (Fig. 4d), which are vasorelaxation inhibitors. Though speculative, these data support the idea that inflammation is reduced in the oral cavity and possibly in the airways as a temporary adaptive mechanism to improve respiratory performance during intense physical activity.

**Fig. 5:**
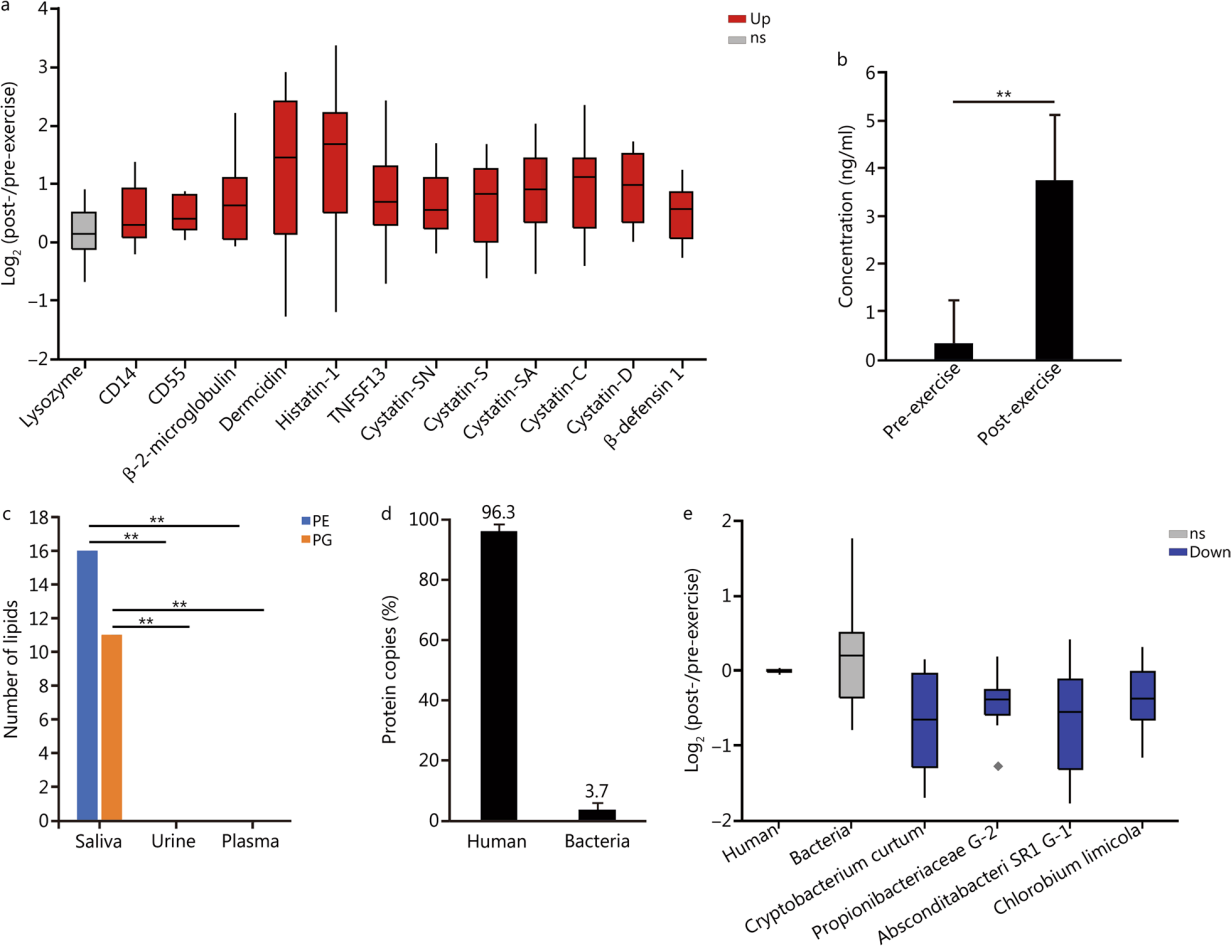
Analysis of the saliva innate immune proteins and microbiota prior and post exercise. **a** Boxplot of abundance ratios of innate immune proteins comparing pre- and post-exercise sections. Stars represent outliers. **b** ELISA analysis of saliva dermcidin levels prior and after the exercise sessions. ^**^P ≤ 0.01 (Student’s *t*-test). **c** Number of diacylglycerophosphoethanolamine and diacylglycerophosphoglycerol species containing C15 fatty acids in different body fluids. ^**^P ≤ 0.01 (Fisher’s exact test). **d** Fraction of human and bacterial proteins in the saliva proteomics. **e** Boxplot of abundance ratios of total iBAQ scores from proteins of different organisms comparing pre- and post-exercise sessions. Stars represent outliers. Down significantly down-regulated molecule, PE diacylglycerophosphoethanolamine, PG diacylglycerophosphoglycerol, Up significantly up-regulated molecule, ns non-significant

We hypothesized that the anti-inflammatory processes might influence susceptibility to infection and trigger a compensating change in other immune system elements. To address this hypothesis, we investigated levels of other immune proteins. Several innate immune proteins were consistently up-regulated after exercise: CD14, CD55 and all antimicrobial peptides/proteins (dermcidin, cystatins, β-defensin 1 and histatin-1) (Fig. 5a). We validated the levels of dermcidin by ELISA. Dermcidin had a 2.5-increase in the proteomics (*P* = 0.01) and a 10.7-fold increase by ELISA (*P* < 0.001) (Fig. 5a, b). The discrepancy in fold change might be due to the levels of dermcidin being close to the detection limit in the pre-exercise sample. This increase in antimicrobial peptides in saliva supports our hypothesis of a compensatory mechanism to improve host defense. We next explored whether there was a functional impact on the oral microbiota. Compared to plasma and urine, we found higher levels of C15 fatty acyl-containing PE and diacylglycerophosphoglycerol (PG) (Fig. 5c), in saliva than in blood and urine, consistent with the expectedly higher load of bacteria in the oral cavity. We reanalyzed the proteomics data by searching against the human and oral microbiome bacterial sequences. We identified proteins from 142 bacterial genera, and we used the intensity-based absolute quantification (iBAQ) method to estimate the relative protein copy numbers. The saliva proteome was comprised of 96.3% human and 3.7% bacterial proteins by copy numbers (Fig. 5d). We used the same quantification to calculate the saliva protein fraction from each organism and compared prior and after the exercise. The exercise session had no effect on the human proteins and the total bacterial proteins, but decreased the abundance of the bacteria *Cryptobacterium curtum*, Propionibacterieae G-2, Absconditabacteria SR1 G-1 and *Chlorobium limicola* (Fig. 5e). The decreases in these four oral cavity bacteria are consistent with our hypothesis that increases in salivary antimicrobial peptides influence oral microbial populations and potentially host susceptibility.

Overall, we observed molecular signatures of down-regulation of inflammation in the saliva, which can be an adaptive response to exercise that improves respiration and blood flow. We also found evidence that the decrease in inflammation is accompanied by an increase of antimicrobial peptides, which altered oral microbiome composition.

## Discussion

Our study illustrates the promise of utilizing advanced mass spectrometry for the global characterization of proteins, lipids, and metabolites to understand the coordinated physiologic and immunologic acute response to intense exercise. In plasma, we observed molecular signatures consistent with those reported in previous studies despite differences in the exercise regimen and cohort characteristics divergent enough to have a significant influence on tissue responses to exercise [5, 11]. Lewis et al. [11] performed a pre- and post-exercise plasma metabolomics analysis of marathon runners, individuals that were submitted to an acute exercise regimen or running a marathon, whereas Contrepois et al. [5] performed a multi-omics analysis of plasma after volunteers reached “peak oxygen consumption” in a short and intense exercise session (8–12 min exercise). In both studies, the average age was in the 50 s, whereas in our cohort the average age was in the 20 s. Like what was reported by Contrepois et al. [5], plasma from volunteers in our study showed signatures of tissue damage and regenerative response after exercise. In terms of metabolism, all three studies showed increased lipolysis [5, 11]. We found that short-chain saturated fatty acids were consumed first, consistent with previous findings [5]. Longer and unsaturated fatty acids were metabolized more slowly, except for C18:1 and C18:2, which are rapidly metabolized like the short saturated fatty acids. Short- and mid-chain fatty acids can be metabolized faster as a result of their carnitine-independent transportation into mitochondria [37]. Unsaturated fatty acids are harder to metabolize due to the requirement of additional beta oxidation steps in the mitochondria [38]. Beta oxidation is accompanied by an increase in the TCA cycle activity. Simultaneously, we observed molecular markers of an increase in glycolysis metabolites, evidenced by the accumulation of its products lactate and pyruvate, but the minimum change in the levels of glucose. Stable levels of plasma glucose are likely the result of a homeostatic mechanism that mobilizes glucose from glycogen stores [11]. Another mechanism could be via dermcidin. Although, dermcidin is mainly characterized as an antimicrobial molecule, more recently it was suggested to play a role in glucose homeostasis by reducing insulin secretion [39].

A major difference between our study and the studies by Lewis et al. [11] and Contrepois et al. [5] was the magnitude of amino acid consumption. While the two prior studies showed that amino acid degradation occurs during the exercise session, this was not evident in our study, which we attribute to differences in age, exercise regimen and training of the cohort. Younger and well-trained individuals have lower amino acid demands during exercise [40]. In addition, amino acid metabolism is not altered in resistance exercise [40]. The increase in alanine and glutamate observed in our analysis is probably due to transamination of the overflowing glycolysis and TCA cycle metabolites pyruvate and α-ketoglutarate [41]. In a parallel study, we analyzed the effects of heat stress on a moderate exercise regimen in which participants walked on the treadmill for 1 h at 5 km/h. We found that only lipid metabolism was regulated during this exercise and no significant difference was observed in glycolysis or amino acid metabolism, when we compared post- and pre-exercise results (unpublished observations). This further supports that exercise regimen can differentially regulate different metabolic pathways.

Increased metabolism supporting the energy demands of exercise leads to higher production of catabolites that must then be excreted in the urine. We found increased levels of the glycolysis products (lactate and pyruvate) and ATP catabolites (inosine, xanthine, and hypoxanthine) in the post-exercise urine. Consistent with our data, these metabolites have been reported to increase after different exercise regimens [42–44]. Stable plasma levels of cystatin C and creatinine were consistent with unchanged glomerular filtration rates after the firefighter training regimen. Others have reported stable glomerular filtration rate even during extreme physical activity, such as running an ultramarathon [45]. We found increases in molecular markers of the renin-angiotensin system in the urine, but not in plasma and saliva. Two other exercise regimens, high-intensity intermittent exercise and moderate-intensity continuous exercise, have both displayed an increase in levels of angiotensin II and angiotensin 1–7 in urine but not in plasma [46]. This is consistent with up-regulation being isolated to the proximal tubule rather than associated with other sources like the liver [47]. Angiotensin is produced in the proximal tubule of the kidney, which may explain the observed increase in urine. Angiotensin II increases the expression of sodium-glucose cotransporters, increasing renal reabsorption of sugars and water [48]. The increased angiotensin II produced in the kidneys may cause vasoconstriction as shown in rats [49]. Conversely, there is vasodilation in the muscles during exercise [50–52], which may help diverge the blood flow and supply nutrients and oxygen to the contracting muscle.

Moderate aerobic exercise has also been shown to improve respiratory mechanics and possibly lung function [53]. We found that the decrease in inflammatory markers was accompanied by increased opiorphin, a peripheral tissue vasodilator [54] that may increase blood flow to muscles during the exercise regimen to improve the delivery of oxygen and nutrients [52]. Our saliva analysis showed a decrease in abundant cellular proteins, which might be due to a reduction in normal epithelial cell turnover in the oral cavity [55]. Nonetheless, moderate aerobic exercise has been shown to decrease eosinophilic inflammation in a murine model of asthma [56]. Airway inflammation caused by allergies, asthma, or chronic obstructive pulmonary disease (COPD) can lead to bronchoconstriction, airflow obstruction and decreases in lung function [57]. We postulate that the decrease in inflammatory molecules we observed in the saliva after exercise might represent an adaptive mechanism to improve gas exchange in response to higher cellular oxygen demand [58, 59]. Furthermore, during strenuous activity, dominant nasal breathers will switch to oronasal breathing at an approximated 35 L/min threshold [60, 61]. At maximum minute ventilation, nasal breathing accounts for approximately 60% of total ventilation; suggesting that the crucial functions of nasopharyngeal filtration, warming and humidification are reduced. Oronasal breathing allows for greater distal airway deposition of microbes, allergens and toxic/irritant particles as mucociliary nasopharyngeal membranes are bypassed [62]. In addition, inhaled air is drier and cooler, which may increase the risk for epithelial cellular damage, encumber mucociliary escalator function, and increase infection susceptibility as an intact epithelial barrier is essential for preventing respiratory infections.

Decreased proinflammatory markers in the oral cavity might also impact the ability to fight against infections. Indeed, marathon runners are at a higher risk for acquiring upper respiratory tract infections after races [63]. We found that decreases in the proinflammatory response occurred alongside increases in antimicrobial peptides and CD14 within the oral cavity. We interpreted these changes as evidence of a compensatory mechanism to maintain immune surveillance. The reduction of specific microbes from the oral microbiome may be a consequence of this increase in antimicrobial peptides. In fact, increases in salivary antimicrobial peptides have been previously linked to prolonged exercise. However, this increase in antimicrobial peptides had no effect on inhibiting *E. coli* growth [64], suggesting a limited capacity of antimicrobial peptides within the oral cavity to protect against host infections. It is plausible that our observed increase in antimicrobial peptide expression was insufficient to compensate for the expected reduction in immune surveillance in the presence of the decreased proinflammatory response.

To study potential impacts on the saliva immune molecule profiles in response to the high-intensity of physical activity we performed a systematic literature review. We queried PubMed for papers that studied respiratory infection events in response to a high-intensity physical activity (see Table 1 for keywords), which included firefighters, soldiers, marathon runners and soccer players. Only papers that performed a survey of respiratory illnesses were qualified for the systematic review. Gaughan et al. [65], monitored a group of 58 wildland firefighters post-fire and compared respiratory questionnaire results to pre- and post-fire season responses. They found that respiratory symptom scores were significantly higher post-fire compared to pre-season and post-season. Similarly, Tiollier et al. [66] performed medical examinations and monitored salivary immunoglobulin A (IgA) levels for a group of 21 soldiers throughout an intense period of training. They found that over 66% experienced at least one upper respiratory tract infection episode which rapidly resolved 2 d after the training session. Robson-Ansley et al. [67], Peters et al. [68], and Nieman et al. [69] followed up on post-marathon runner upper respiratory symptomology via questionnaire. All three studies found at least twice higher incidence of self-reported upper respiratory infections compared to control groups. However, Robson-Ansley et al. [67], attributed respiratory symptomology to elevated immunoglobulin E (IgE) serum levels, suggesting that respiratory symptomology was due to eosinophilic inflammation rather than neutrophilic inflammation. Furusawa et al. [70] found no significant differences in upper respiratory tract infection rates in a cohort of wheelchair marathon racers. Conversely, Moreira et al. [71] monitored salivary IgA, salivary cortisol, and upper respiratory tract infection symptoms within a cohort of 34 soccer players during practice season, competition and post-season. They found that the number of upper respiratory symptoms significantly reduced, while salivary IgA significantly increased 2 weeks after the competition season ended. They concluded that intense training and competition may attenuate mucosal immune responses and neutrophilic inflammation. In summary, there is evidence supporting a relationship between physical demands and a higher incidence of respiratory infections.

Our study had limitations that potentially reduced the generalizability of our results. Study participants had a relatively low diversity being historically healthy, athletically fit, young adult males. They were chosen from an available small sample of local wildland firefighters. The study did not include women, which is a reflex of the firefighter population in the US which is composed of 96% males [72]. The small sample size might bring concerns of findings statistical significance and interference with confounding factors, but the “within subject” differential expression before and after intense exercise reduces the impact of the sample size. In addition, we estimated that the study was sufficiently powered for its intended aims. Increasing the number of subjects could increase the number of identified lipids, proteins, and metabolites, but would not change the main conclusions of the paper. Moreover, a metabolomics analysis of plasma showed strikingly similar profiles and trends in terms of lipolysis, glycolysis, TCA cycle, tissue damage and regeneration response when compared to other exercise metabolomic studies [5, 11]. Unlike Contrepois et al. [5], our exercise protocols were not standardized, and peak oxygen consumption (VO_2_ max) was not measured to monitor whether participants reached their maximal exercise capacity during the 45 min exercise session. However, the accumulation of lactate is a reliable indicator of anaerobic metabolism, suggesting that exercise intensity had surpassed aerobic respiratory capacity in most of our participants [73]. It is also important to note that clinical trials should be carried out before using these findings for human health.

Another overriding limitation would be the generalizability of potential health impacts to the general population or other elite athlete exercise multi-omic studies because of the characteristics of the different populations. Nonetheless, when we compared our results to those considered in the systematic review cohorts, we did observe a similar trend regarding possible increased susceptibility to respiratory infection after intense physical activity. Unfortunately, four of the seven studies examined, relied upon self-reported respiratory symptoms to indicate respiratory infection incidence. While self-reporting through questionnaires is a common method to collect health information, it may not be the most accurate or consistent method. The presence of respiratory symptoms does not differentially diagnose respiratory infection. Additionally, self-report of symptoms is subject to recall bias and makes it more difficult to obtain consistent outcomes across a study because respiratory symptoms also occur during eosinophilic inflammation or hypersensitivity reactions. However, one of the reviewed studies considered the presence of fever obtained during medical examination, as well as respiratory symptoms to diagnose respiratory infection. This helped to reduce bias and disease misclassification [68].

One final limitation that may be unique to wildland firefighters, is their perennial exposure to specific respirable toxic pollutants during fire suppression activities [74, 75]. These individual environmental exposures may permanently alter immune system immunomodulation and gene expression of key metabolic pathways to confound our results. Further epigenetics investigation would help to illuminate this potential bias.

## Conclusions

Overall, we observed clear evidence for the regulation of physiological and biochemical processes typically coordinated to supply energy and oxygen demands from intense exercise. In plasma, we found signatures of tissue damage and an acute repair response. In terms of metabolism, lipolysis, glycolysis, and TCA cycle were up-regulated to meet increased energy demands. The urine analysis showed a strong regulation of the renin-angiotensin system toward an adaptive response required to increase catabolite elimination, reabsorption of nutrients and maintain fluid balance. Decreases in inflammatory molecules and increases in antimicrobial peptides in saliva could be viewed as paired responses, one to improve respiratory function, and the other to increase immune surveillance under conditions of increased susceptibility to infection. Furthermore, we also found that this compensatory mechanism might be insufficient to protect professionals after tasks that demand intense physical effort. This study is a critical first step towards developing a comprehensive understanding of the physiological, biochemical, and immunological effects of stress, definition of the molecular markers of those effects, and the development of effective methods for monitoring and mitigating those effects in critical first responder populations like firefighters.

### Supplementary Information

**Additional file 1:** **Table S1** Quantitative proteomics analysis of plasma, saliva, and urine. Plasma samples were immunodepleted for the top 14 most abundant proteins, digested with trypsin, labeled with tandem mass tags, and fractionated by high pH reverse phase chromatography before being analyzed by LC-MS/MS. Saliva and urine were digested with trypsin and analyzed by label-free LC-MS/MS. Proteins were considered significantly different with a *P* ≤ 0.05 by Student’s *t*-test (highlighted in green). Fold changes are highlighted in degrees of red (up-regulation) and blue (down-regulation). **Table S2** Quantitative lipidomics analysis of plasma, saliva, and urine. Plasma, saliva, and urine extracted with chloroform: methanol: water and analyzed by LC-MS/MS in both positive and negative ionization modes. Lipids were considered significantly different with a *P* ≤ 0.05 by Student’s *t*-test (highlighted in green). Fold changes are highlighted in degrees of red (up-regulation) and blue (down-regulation). “_A”, “_B” or “_C” denotes different chromatographically resolved isomers. **Table S3** Metabolomics analysis of plasma, saliva, and urine. Plasma, saliva, and urine extracted with methanol, derivatized and analyzed by GC-MS. Metabolites were considered significantly different with a *P* ≤ 0.05 by Student’s *t*-test (highlighted in green). Fold changes are highlighted in degrees of red (up-regulation) and blue (down-regulation). **Table S4** Quantitative plasma proteomics analysis. Plasma samples were immunodepleted for the top 14 most abundant proteins, digested with trypsin, labeled with tandem mass tags, and fractionated by high pH reverse phase chromatography before being analyzed by LC-MS/MS. Significance was calculated with Student’s *t*-test. Values represent normalized intensities. **Table S5** Quantitative plasma metabolomics analysis. Plasma samples were extracted with methanol, derivatized with *N*-methyl-*N*-(trimethylsilyl)trifluoroacetamide and analyzed by GC-MS. Significance was calculated with Student’s *t*-test. Values represent normalized intensities. **Table S6** Quantitative plasma lipidomics analysis. Plasma samples were extracted with MPLEx and analyzed by LC-MS/MS in both positive and negative ionization modes. Significance was calculated with Student’s *t*-test. Values represent normalized intensities. **Table S7** Quantitative urine proteomics analysis. Urine samples were digested with trypsin and analyzed by LC-MS/MS. Significance was calculated with Student’s *t*-test. Values represent normalized intensities. **Table S8** Quantitative urine metabolomics analysis. Urine samples were extracted with methanol, derivatized with *N*-methyl-*N*-(trimethylsilyl)trifluoroacetamide and analyzed by GC-MS. Significance was calculated with Student’s *t*-test. Values represent normalized intensities. **Table S9** Quantitative urine lipidomics analysis. Urine samples were extracted with MPLEx and analyzed by LC-MS/MS in both positive and negative ionization modes. Significance was calculated with Student’s *t*-test. Values represent normalized intensities. **Table S10** Quantitative saliva proteomics analysis. Saliva samples were digested with trypsin and analyzed by LC-MS/MS. Significance was calculated with Student’s *t*-test. Values represent normalized intensities. **Table S11** Quantitative saliva metabolomics analysis. Saliva samples were extracted with methanol, derivatized with N-methyl-N-(trimethylsilyl)trifluoroacetamide and analyzed by GC-MS. Significance was calculated with Student’s *t*-test. Values represent normalized intensities. **Table S12** Quantitative saliva lipidomics analysis. Saliva samples were extracted with MPLEx and analyzed by LC-MS/MS in both positive and negative ionization modes. Significance was calculated with Student’s *t*-test. Values represent normalized intensities.

## Data Availability

The raw LC-MS/MS proteomics data files are publicly available at ProteomeXchange-associated Pride data repository under the accession numbers PXD020122, PXD020131, PXD020164 and PXD020352. All raw lipidomics and metabolomics data files are publicly available at MetaboLights data repository under the accession number MTBLS1891.

## Abbreviations

ACE: Angiotensin-converting enzyme
ACE2: Angiotensin-converting enzyme 2
COPD: Chronic obstructive pulmonary disease
DG: Diacylglycerol
ECM: Extracellular matrix
EDTA: Ethylenediaminetetraacetic acid
GC-IS: Gas chromatography-mass spectrometry heavy isotope internal standard mix
GC-MS: Gas chromatography-mass spectrometry
iBAQ: Intensity-based absolute quantification
IgA: Immunoglobulin A
IgE: Immunoglobulin E
LC-MS/MS: Liquid chromatography-tandem mass spectrometry
LPE: Monoacylglycerophosphoethanolamine
MSTFA: *N*-Methyl-*N*-(trimethylsilyl)trifluoroacetamide
PC: Diacylglycerophosphocholine
PCO: Acylalkylglycerophosphocholine
PE: Diacylglycerophosphoethanolamine
PG: Diacylglycerophosphoglycerol
PI: Diacylglycerophosphoinositol
rMd-PAV: Robust Mahalanobis distance based on peptide abundance vectors
SM: Sphingomyelin
SPANS: Statistical procedure for the analyses of peptide abundance normalization strategies
TCA: Tricarboxylic acid
TG: Triacylglycerol
TMCS: Trimethylchlorosilane
TMT: Tandem mass tags
VO_2_ max: Peak oxygen consumption
